# The body cannot be cheated: sexual practices and modern contraceptive use among street-involved young people in two South West States in Nigeria

**DOI:** 10.12688/aasopenres.13241.1

**Published:** 2021-08-04

**Authors:** Mary O. Obiyan, Atinuke O. Olaleye, Macellina Y. Ijadunola, Morenike O. Folayan

**Affiliations:** 1 Demography and Social Statistics, Obafemi Awolowo University, Ile Ife, Nigeria; 2 Department of Obstetrics and Gynecology, Babcock University, Ilishan, Nigeria; 3 Department of Community Health, Obafemi Awolowo University,, Ile-Ife, Nigeria; 4 Department of Child Dental Health, Obafemi Awolowo University, Ile-Ife, Nigeria

**Keywords:** Street Youths, Young People, Sexual and Reproductive health, Sexual Practices, Risky Behaviour, Transactional Sex, Modern Contraceptives, Nigeria

## Abstract

**Background**: Young people aged 10–24 years constitute about one-third of the total population of Nigeria. Street-involved young people (SIYP) face a double burden of living condition instability and lack of adequate parental monitoring. This leaves them vulnerable to poor sexual and reproductive health (SRH) choices and behaviour. Risky sexual behaviour with poor access to SRH information and interventions increases their vulnerability to adverse SRH outcomes. This study explored the use of modern contraceptives and sexual practices among male and female SIYP (10–24 years) in Nigeria.

**Methods:** This qualitative study used an exploratory research design to guide the development of the focus group discussion (FGD) and in-depth interview (IDI) guides. Participants were asked questions on background information, lived experiences and SRH practices. The FGDs were stratified by sex and age. Both FGD discussions and IDI interviews were recoded; transcripts were transcribed and translated from local dialect into English language. Content analysis was conducted thematically with the aid of NVivo.

**Results:** In total, 17 IDIs and 11 FGDs were conducted among SIYP aged 10–24 years. The total number of respondents interviewed was 109. There is high awareness of modern contraceptives among SIYP; the commonly known method was condom with a few also aware of emergency contraceptives pills. However, participants reported low use of modern contraceptives. The common reasons alluded for not using condoms were reduced sexual pleasure, cost and associated myths. The five themes that emerged under sexual practices of SIYP included early age at first sexual encounter, multiple sex partners, transactional sex, same-sex relationships, and transactional sex.

**Conclusions:** There is low utilization of contraceptives by SIYP against the background of their high-risk sexual practices. SIYP would benefit from free contraceptive education, social safety nets and interventions to dissuade them from transactional sex and other unhealthy sexual practices.

## Introduction

Adolescents and young people have a heightened tendency for risky sexual practices such as early age at sexual debut, having multiple sexual partnerships, unprotected sexual intercourse and other risky behaviours, which are associated with the risk of contracting human immunodeficiency virus (HIV) and other sexually transmitted infections (STIs), and having unwanted pregnancies ^1, 2^. Factors such as lack of parental support ^3^, peer pressure, idleness, socioeconomic status, unfriendly socio-cultural norms, lack of school connectedness ^4^, religiosity ^5^ and low level of education ^6^, increase the probability of adolescents taking these risks. Despite the increasing sexual activities of young people, few of them use a modern contraceptive method ^6, 7^. HIV disproportionately affects young people and adolescents [10–19 years] in Nigeria, as the prevalence of 3.5% is the highest among West and Central African countries and a major cause of death among male adolescents ^6, 8^.

Unwanted pregnancy is a major reproductive health problem for adolescents and young people, especially in low and middle-income countries, exacerbated by low uptake of modern contraceptives ^9, 10^. Contraception use can significantly reduce the incidence of unwanted pregnancy and other associated negative health outcomes of risky sexual practices. However, public support for access and use of contraception by adolescent boys and girls is very low as adolescents’ sexual intercourse and contraceptive use are perceived as indecent ^8^.

There is a wide gap between the knowledge of modern contraceptives and its utilization in Nigeria, with knowledge being higher than its use ^11^. The 2018 Nigeria Demographic Health Survey (NDHS) reported condom use knowledge to prevent HIV among women of ages 15–19 years, 20–24 years and 25–29 years as 64.6%, 75.1% and 75.3%, respectively ^12^. Similarly, the modern contraceptive prevalence rate was given as 2.3% (15–19 years), 8.2% (20–24 years) and 12.3% (25–29 years) ^12^. The factors associated with low modern contraceptive prevalence rates include technical and interpersonal skills, provider bias, erratic supply of contraceptives, type of facility, socio-demographic and economic factors such as age, education, parity, urban residence, wealth, perceived self-efficacy to take contraceptives, social approval of contraception, provider-imposed restrictions and misconception about modern contraceptives ^11, 13, 14^. Some other factors are informed choice and costs of contraceptives ^15^.

Street-involved young people (SIYP) are more vulnerable to risky sexual practices ^16^. An Ethiopian study showed that over 60% of street youth reported early age at sexual initiation, transactional sexual practices and having multiple sexual partners due to high exposure to sexual exploitation and unstable lifestyles ^16^. Substance use is also a risky behaviour identified among SIYP. Evidence has associated substance use with having unprotected sex and multiple sexual partners ^17^. SIYP are vulnerable and stigmatized, contributing to an unstable life, unhealthy practices and risky behaviour ^16, 18, 19^. Limited knowledge and access to SRH services including modern contraceptives, make this vulnerable group take sexual risks such as sexual intercourse with no condoms, prostitution and trading sex for food or protection to survive ^19, 20^.

Despite the sexual risk-taking behaviours of SIYP, many health programs exclude this vulnerable population subgroup contributing to adverse SRH outcomes. However, limited information on the SRH of street-involved young people in many African contexts, including in Nigeria, was attributed to the inequalities in sexual health interventions. Where some information exists, it is either limited to gender, mostly female, or a specific age group. Therefore, this study explored the perspective of male and female SIYP about modern contraceptive knowledge and use against the background of their sexual practices.

## Methods

### Ethical approval

Ethical approval for this study was obtained from the Institute of Public Health Research Ethics Committee, Obafemi Awolowo University Ile-Ife (IPHOAU/12/1133). Additionally, ethical approval was obtained from Osun State (OSHREC/PRS/569T/154) Health Research Ethics Committee, and, social approval (LSMH2695/11/260/T) from the Lagos State Ministry of health. The study was conducted in line with the declaration of Helsinki. Written consent was obtained from all study participants. Parental consent was waived for children below 18 years from all the ethics committees. This was because the study was non-invasive, the children were street kids, many being matured minors, and the associated challenges of locating parents for study approval purposes.

### Study design

This qualitative study is a part of a concurrent mixed-method survey study designed to examine and explore the SRH of SIYP. The study recruited respondents using respondent-driven and time-location sampling methods ^19^ from clusters where SIYP gather in large numbers. The selected areas were Bariga and Ajah in Lagos State and Oke-Baale, Olaiya and Sabo in Osun State. The respondents recruited for focus group discussions (FGD) or in-depth interviews (IDI) were identified during the survey ^19^. Male and female field workers were trained to identify eligible participants for the IDI and FGD, respectively. The qualitative study followed the consolidated criteria for reporting qualitative research (COREQ) specified guidelines for conducting and reporting qualitative research ^21, 22^. The completed COREQ checklist can be found as extended data ^23^. An exploratory approach guided the interview guide’s development to explore perspectives and behaviours of SIYP concerning their SRH. The qualitative data was collected through focus group discussions (FGDs) and semi-structured in-depth interviews (IDIs) using digital audio recorders. Data was collected between January to February 2019.

### Study population

This study participants were male and female SIYP aged 10–24 years in two urban communities in south-west Nigeria. The eligibility criteria entailed living ‘on’ (returns home at night) or ‘of’ (never returns home) the street in Lagos or Osun state and being mentally stable ^19^. Interviewees were informed of the FGD and those willing to participate were implored to reconvene shortly after the survey for the session. For IDI, the field workers were earlier trained during the interview-administered questionnaire to identify participants who answered in the affirmative to some or all of the questions as indicators of risky sexual practices. This included transactional sex, multiple sexual partners, inconsistent use of condom, pregnancy and induced abortion for the female SIYP, self-reported depression or had attempted suicide.

The fieldworker informed the supervisors of the possible IDI participants, who then followed up with the SIYP through one-to-one discussion to seek consent for recruitment and voluntary participation. Thus, all participants for the FGD and IDI were purposively selected based on responses to questions asked during the survey. The time and venue for the interview was also scheduled. The community meeting centres was used as the venue for all the FGD sessions which took place at different times. The IDI participants were given a snack and soft drink during or after the interview. A token of $0.56 was provided as transport fare to those who used “okada” (motorbike), the common mode of transport within an area or street to street) to the venue of data collection.

### Study procedure

Respondents for the FGD were asked to return for the exercise after completing the survey and were assembled at a comfortable place away from distractions. The FGD was stratified by age (10–14, 15–19, 20–24 years) and gender (male, female) of study participants in each of the study areas to allow ease of communication during discussions. However, only five FGDs were conducted in Lagos State due to constant disruptions resulting from street gangs’ clashes in Bariga – a central study area in the State. Six FGDs were held in Osun State. There were between 8 and 10 participants in each FGD session moderated by a trained interviewer and a note-taker. A total of 8 research assistants with skills and experience of collecting qualitative data facilitated the FGD sessions. All the FGD participants assumed pseudonyms for identification purposes during the session to maintain strict confidentiality of their original names. Assuming pseudonyms in qualitative research has been well practiced, creating a friendly and mutual platform for both participants and moderator.

The IDI was conducted for respondents who consented to participate at a comfortable and secluded location away from other SIYP for privacy. For adolescents 10–19 years of age, a male interviewer conducted the IDI for a male participant and a female interviewer interviewed a female participant. For young person’s 20–24 years of age, either sex conducted interviews for male and female participants. There was no participant that declined participation in the FGD and IDI. Discussions and interviews were audio-recorded, and notes taken. The local language (Yoruba), pidgin English and Hausa dialect were used to conduct the interviews. All FGD and IDI sessions conducted in the local dialect were translated to English for analysis purposes.

Research assistants skilled in transcribing and analysis of qualitative data double-checked the translations to ensure meanings and context were retained. A PhD graduate student who speaks and writes Hausa language checked the translations of the Hausa recordings to ascertain that no meaning was lost. None of the transcripts was returned to participants for verification due to their constant movement and low educational status. The IDI session was supervised by MOO and interviewers were trained to be non-judgmental, friendly, emotionally intelligent and conscious of the need to pause, stop or reschedule an interview if need be. Debriefing meetings were held during the conduct of study among fieldworkers, supervisors and research team to discuss issues arising during the interview sessions.

Participants were briefed on the study aims and objectives and informed of their right to withdraw at any phase of the study before the IDI or FGD sessions began. Written and verbal consent were obtained from participants. The interviewers had no previous relationship with the study participants. The FGD and IDI were conducted to explore the knowledge and use of modern contraceptives, sexual practices, beliefs.

The IDI and FGD guides focused on specific areas: (a) background information, (b) lived experiences as a street child or youth – challenges and resiliency (c) SRH – perceptions and practices, (d) sexually transmitted diseases, (e) substance use (f) illness and disabilities., and (g) future life plans. The guides can be found as extended data ^23^.

### Data

Data for this study were based on the transcripts of 11 FGDs (4 for male participants, 7 for female participants) and 17 IDIs (8 male and 9 female participants). The time range for the FGD was 30 to 50 minutes while that for the IDI was between 37 and 42 minutes. This study’s qualitative data was collected concurrently alongside the quantitative data between January to February 2019. 

### Analysis

 The transcribed data was imported into NVivo 11 to aid analysis. After that, inductive line-by-line coding was used to build and explore themes emerging from the transcripts, as this allows for themes and concepts to be inductively recognized ^24^. A research team member (MOO) supervised and worked with a team of 6 graduate students during this phase for theme extraction and categorization. New emerging codes were submerged into existing ones to form a broader theme, created as a sub-code or form a new category. Coding stopped when saturation was attained, defined as when no new codes emerged ^25^. Content analysis was conducted thematically with the aid of NVivo 11– a software program for qualitative analysis.

## Results

The background characteristics of the study participants are highlighted in Table 1. The mean age for female participants was 17.6 years, and for male was 19.4 years. Most of the FGD participants were female (60, 65%), aged between 15–19 years (45, 49%) and reported to be unemployed (69, 75%). The IDI participant characteristics revealed that they were mostly (9, 53%) females, aged 20–24 years (10, 59%) and unemployed (11, 65%). Across both FGD and IDI participants, there were more with incomplete secondary education (54, 50%), practicing the Islamic religion (69, 63%), single (102, 93.6%) and reside in Osun state (66, 61%). Common jobs reported were tailor, shoe-maker, hairstylist, street-parker, sand packer, bus conductor, photographer, sex worker, fish trader and welder.

**Table 1.** Characteristics of focus group discussion (FGD) and in-depth interview (IDI) participants.

Characteristics	*FGD Participants (N = 92)	IDI Participants (N = 17)	Total (N = 109)
**Gender**			
**Female**	60 (65.2%)	9 (52.9%)	69 (63.3%)
**Male**	32 (34.8%)	8 (47.1%)	40 (36.7%)
**Age (years)**			
**10–14**	12 (13.0%)	2 (11.8%)	14 (12.8%)
**15–19**	45 (49.0%)	5 (29.4%)	50 (45.9%)
**20–24**	35 (38.0%)	10 (58.8%)	45 (1.3%)
**Completed Education **			
**None**	1 (1.1%)	---	1 (0.9%)
**Incomplete primary**	5 (5.4%)	2 (11.8%)	7 (6.4%)
**Completed Primary**	30 (32.6%)	1 (5.9%)	31 (28.5%)
**Incomplete secondary**	45 (48.9%)	9 (52.9)	54 (49.5%)
**Completed secondary**	11 (12.0%)	5 (29.4%)	16 (14.7%)
**Work Status**			
**Employed**	23 (25.0%)	6 (35.3%)	29 (26.6%)
**Unemployed**	69 (75.0%)	11 (64.7%)	80 (73.4%)
**Marital status**			
**Single**	88 (95.7%)	14 (82.4%)	102 (93.6%)
**Married**	4 (4.3%)	3 (17.6%)	7 (6.4%)
**Religion**			
**Christianity**	31 (33.7%)	9 (52.9%)	40 (36.7%)
**Islam**	61 (66.3%)	8 (47.1%)	69 (63.3%)
**Location**			
**Osun state**	57 (62.0%)	9 (52.9%)	66 (60.6%)
**Lagos state**	35 (38.0%)	8 (47.1%)	43 (39.4%)

* 11 FGDs = 7–10 Participants in each FGD.

### Findings from FGD and IDI

 During the FGD sessions, questions about sexual practices, knowledge and use of modern contraceptives were asked. Questions were also asked on the lived experiences and survival on the streets among the SIYP. The themes that emerged were perspectives, myths and use of modern contraceptives, individual and community sexual practices including age at first sexual intercourse, multiple sexual partners, pregnancy and abortion, transactional sex and same-sex sexual practices. The emerged themes were thereafter categorized into three: (i) awareness and use of modern contraceptives, (ii) perspectives and myths about modern contraceptive, and (iii) sexual practices. Sub-themes of sexual practices of SIYP were age at sexual debut, multiple sexual partners, transactional sex, and same-sex sexual relationship. While the perspectives mentioned in the above themes ranged from individual to socio-cultural issues, they do, however, vary by participant’s age, gender and extent of street involvement. 

### Theme 1: Awareness and use of modern contraception

This study explored the awareness and use of modern contraceptives among SIYP. Many male and female participants were aware of condoms. The girls were aware of pills and emergency contraceptives (postinor). Many mentioned concoctions as modern contraception. There was low awareness of other contraceptive methods such as female condoms, intra-uterine devices and injectables. This indicates poor knowledge of contraception among SIYP, which may explain the high risk of unwanted pregnancy among the group.

*“I do hear about something like postinor, I think they said once they are through with sleeping with each other, they use it, so that they won’t get pregnant. That’s the one I hear very well. Other things the young ones use to avoid pregnancy are condoms” (21-year-old Female IDI participant)*
*“Alabukun (analgesic) is the form of contraception that I use. I’ll quickly pour it inside water. Alabukun works. I might also use postinor, because before 3 days, you need to do something. You don’t know if the person actually discharged on your body” (20-year-old Female IDI participant)*

Condoms and pills were used inconsistently during sexual intercourse, and many perceived the use of condoms in stable relationships as unnecessary. When a condom was used, it was to prevent pregnancy and STIs. Poor access to SRH services was a reason identified for inconsistent use of condoms. Cost was a limiting factor for accessing condom: although the purchasing cost of condoms was low – less than $1 per pack (3pcs)– this was still not affordable for many SIYP. Participants reported they barely survive on the street, hence had little to spare to purchase condom.

One participant identified that condoms were used when partners distrust themselves. Limited knowledge of other modern methods also accounts for the low usage of contraception.

*“people are using condom but you know … those that don’t use condom they have boyfriend and those that don’t have they use” (20-year-old Female FGD participant)*
*“Once condom is distributed, people will start using it widely. I mean distributed freely, because as cheap as it is some people do not have the means to buy it.” (19-year-old Male FGD participant)*

### Theme 2: Perspectives and myths about modern contraceptives

There were myths about contraceptives one of which is that condoms are only used by immoral people, such as “runs girls” (a local name for female sex workers), or drug addicts. Some believe that condoms are a source of diseases to the user and are only to be used when a partner has a disease. The myths associated with the use of condoms reported by the study participants are listed in Table 2.

**Table 2.** Quotes by participants describing myths about condoms. FGD=focus group discussion.

Myth	Excerpts
People who have “boyfriend” or “girlfriend” don’t use condom	*“people are using condom but you know, people that don’t use condom are those that have boyfriend and me I don’t have boyfriend* *so any one that comes my way I will use condom”* (24-year-old, Female FGD participant)
Use of condom brings diseases	*“There are some that said they do not like condoms during sex, while there are some who prefer the use of condom. You know our * *body make-ups are different, there are some that cause diseases to one’s body and there are some that do not* “(20-year-old Female FGD participant)
The oil in condom causes infection	*“the oil in condom always cause infection, the infection scratch a woman because when you use the condom on your private part, it* * will be scratching you, when you open a condom you will see the oil you understand when you put it in your body, the condom will be * *scratching you so when I bring it out I will use tissue and clean it, I will change the oil and then I apply my own cream”* (16-year-old FGD female participant)
Condom causes pain to the body	*“when the condom is dry the condom will now start touching the skin directly the person will be having pain and that is how infection* * start*” (21-year-old FGD female participant) *“I don’t use any form of contraception, even ladies now a days don’t like it. They say it pains them below, like peppers them* ...” (21-year- old Male FGD participant)
Condom is used by immoral persons, “runs girls” and drug users	*“what I know about condom is that those that are called ‘RUNS GIRLS’ uses it”* (18-year-old Female FGD participant) “ *condom is being used by those who smoke marijuana”* (12-year-old Female FGD participant) “ *about condom … it’s only an immoral person that would be using it. It’s usually immoral people that use it, it’s when a man is immoral* * that he would use it, when a woman too is immoral, she would use condom*” (24-year-old Female FGD Participant)
Condom is used only by women who just gave birth to prevent another pregnancy	*“if a woman just gave birth she will be instructed by the doctor to use condom while having sex with her husband after child birth”* (18- year-old FGD female participant) *“those women that just finished giving birth use it to prevent pregnancy”* (20-year-old Female FGD participant)

### Theme 3: Sexual behaviour and practices

This study explored respondents’ perspectives on sexual intercourse, age at first sexual experience, number of sexual partners, same-sex sexual relationships and transactional sex.

***Sub-theme 3.1: Perspective about sexual intercourse***. Male and female participants, including young adolescents (aged 10–14 years), perceived having sex as a norm for having fun, a show of affection and a proof of loyalty to one’s partner. Some others reported sex as an exchange tool for monetary and other transactions

To further explore the perceived importance of engaging in sexual activity, participants were asked if they can practice abstinence – most male and female SIYP answered in the negative. See excerpts below:

*“If it is a youth above eighteen years, then you know, having sex is necessary, the body cannot be cheated (18-year-old Male FGD participant)*
*“no one can do without sex” (16-year-old Female FGD participant)*

***Sub-theme 3.2: Age at first sexual intercourse***. The reported ages at first sex for male and female participants raged from 10 to 15 years. The peer group and rape were among the reported reasons for early sexual debut, especially for the females.

*“For young girls at the age of 13 years that are sexually active, it’s not their joy, the ones that have lost their virginities at that tender age it’s not their happiness. They might have been raped while some willingly gave themselves but majority of them didn’t do it intentionally” (18-year-old Female FGD participant).*

The participant’s views on an appropriate age at sexual debut varied by sex. The male SIYP mentioned early ages for a girl to begin sexual activity, while the female SIYP mentioned later ages which they associated with maturity. Some male SIYP reported girls should begin sexual activity with the commencement of puberty which is assumed to indicate the development of sexual organs.

However, many participants perceived that self-awareness and maturity should define the onset of sexual activity.

*“For a lady if she starts to develop breast she is good to start having sex like from age 14 and for the guy at age 17 to 18 but then it depends on the way you find your life” (22-year-old Male FGD participant)*
*“It’s from the age of eighteen because by then the person will have some knowledge. That person must have been to school, they must have taught her sex, reproductive system, how to prevent sex, how to prevent pregnancy how to prevent infection all this at the age of eighteen” (23-year-old Male FGD participant)*
*“from twenty-three (23) so that she knows what she is doing, she is wise … she can make decisions, for someone who wants to have boyfriend, she will know how to protect herself, she will know if what she is doing is bad, wrong or good (24-year-old Female FGD participant)”*

***Sub-theme 3.3: Multiple sexual partners***. Male and female participants reported having multiple sexual partners at the time of the study. Male SIYP kept multiple sexual partners for pleasure and to prove masculinity, while the female SIYP kept multiple sexual partners for monetary gains.

*“I can’t count the number of sexual partners I have had. I have lost count” (22-year-old Female IDI participant, Lagos)*
*“I have; I have about three… I don’t know why, I just packed them together” (19-year-old Male FGD participant)*

***Sub-theme 3.4 Same-sex sexual relationship***. Few SIYP, mostly male, reported having same-sex sexual relationships. A male SIYP reported engaging in same-sex sexual relationships because he derives no pleasure from sex with females. Another female SIYP reported having engaged in same-sex sexual activity at the command of an older male client. This shows the vulnerabilities of SIYP especially the females while striving for survival and livelihood. Most participants reported to have no interest in same-sex sexual relationship.

“ *Ha … Yes, I do have same-sex affair … I do not fancy females that much” (16-year-old Male IDI participant)*
*“I have had sex with someone of the same sex. My friend and I went to a show where we met this wealthy man. So, he told us, my female friend and I, to first have sex with ourselves. We had sex with ourselves, then we went to meet him. We got the money eventually.” (19-year-old Female IDI participant)*

***Sub-theme 3.5: Transactional sex***. During the interview, transactional sex was defined to respondents as non-marital and non-commercial sexual relationships. This involves an exchange of sex in return of gifts, money, favour, goods or services. Among male and female SIYP, exchanging sex for money, food, clothing and shelter was common. Earning a living to care for self and dependents is a challenge for many SIYP and a reason why some transact sex.

*“I also exchange sex for money, because I have to take care of my younger sibling. He’s living with me. I can’t let him suffer … I also exchange sex for shelter. How much will I need to rent an apartment? I don’t have money.” (20-year-old Female IDI participant)*
*“When I go to meet guys, I need some money or even when I am hungry, I will go and talk to someone I know can feed me, then they start asking you to touch or have sex, I have no choice because I am hungry or what I need is very important… painfully…some will now sleep with me and say they will transfer and it will never come” (20-year-old Female IDI participant)*
*“it’s in the bus we sleep, and those bus drivers don’t give it free, a lot of things we do in exchange for something. So, we call it survival” (19-year-old Female IDI participant) *

## Discussion

This study explored the perspective of male and female SIYP in two South West States in Nigeria about their awareness and use of modern contraceptives and sexual behaviour and practices. The study found that most participants are aware of modern contraceptives. The commonly known method is a male condom which is consistent with findings among homeless youth in other environments ^26^, while most of the participants were unaware of the female condom. Influences for poor condom use were based on personal perceptions of sexual satisfaction and partner’s fidelity, as well as the social perception of immorality among condom users and other myths. Affordability is also a challenge in this group because though condoms are relatively inexpensive, it is not a priority for someone who is trying to survive simply. This portrays the limited accessibility and availability of SRH services among SIYP, an already marginalized population subgroup ^27^.

SIYP have high unmet needs for contraceptives use and other SRH programs. Compared to other young people in the country, the living realities of SIYP make them often left out of programs and interventions that promote awareness, knowledge, and uptake of modern contraception. Due to a combination of societal discrimination, their instability and fluid movement, SIYP remain undocumented and neglected from national initiatives aimed at improving health care and positive SRH behaviours among the youth.

Generally observed in this study is the concordance between individual perceptions of sex and their sexual practices. Common perceptions among participants in this study are the impossibility of sexual abstinence and the necessity to engage in sexual activities regularly for fun and as proof of fidelity to one’s partner. For those who perceive sex as a survival tool, it is practiced transactionally for monetary gains or exchange for basic necessities and services. Myths about condoms may have influenced the common practice of unprotected sex, in addition to the poor negotiating power of this vulnerable group. This is consistent with findings from other studies, as street children are vulnerable to sexual exploitation, which increases their risk for contracting HIV and other STIs ^18, 28^.

SIYP in this study also affirmed the non-use of condoms among sexual partners to show love and proof of infidelity. However, this was commonly expressed among the female SIYP indicating a higher vulnerability than their male counterparts. While the males keep multiple sexual partners and at risk for contracting and transmitting STIs, the female partners at the receiving end has limited negotiating power for safe sex practices. The poor socio-economic status, lack of social support, and stigmatization all contribute to this population subgroup’s vulnerabilities. This accounts for why many SIYP indulge in risky sexual behaviour including transactional sex, multiple sexual partners and early age at sexual debut.

The reported ages at first sexual experience among male and female participants varied, however, early ages were commonly reported ranging from 10 to 15 years. Early age at sexual debut has been identified as a risk factor for unintended pregnancy among adolescents, as they are less likely to practice effective contraception ^1^. The community, peer group and rape were reported as precipitants of early age at sexual debut especially females. The vulnerability of SIYP due to low perceptions of their sexual risk, desperation for survival, poor negotiating power for protected sex, and lack of access to SRH services place them at greater risk of sexually transmitted infections, unintended pregnancy and unsafe abortion. A quantitative study among youth in Uganda found that homelessness was associated with two times higher odds of HIV infection and three times higher odds of experiencing rape ^28^. The participants’ views on an appropriate age at sexual debut differed, as male SIYP mentioned early ages for a girl to begin sexual activity, while female SIYP mentioned later ages which is suggestive of maturity ^1, 19, 29^.

Participants referred to same-sex relationships as a sexual activity randomly engaged in for pleasure or monetary gains while very few mentioned it as a norm and acceptable sexual behaviour. The societal perception of homosexuality may have contributed to fewer participant’s disclosure especially during the FGD sessions.

Having multiple sexual partners was commonly affirmed by many participants. This further corroborates the many risky sexual behaviour of SIYP. The alluded reasons were for pleasure and as proof of masculinity for the male SIYP. On the other hand, it was for monetary and survival reasons for most female SIYP. Across different gender and ages, giving sex in exchange for money, food, clothing and shelter were common practices, as survival and earning a living on the street remained a challenge for many SIYP ^30^. Female SIYP reported engaging in transactional sex as a norm to meet daily needs, while for many male SIYP, it was mostly practiced for monetary reasons only. In the many excerpts of participants, the most common reason for transactional sex was shelter and money. Thus, socio-economic reasons play a major role in determining sexual practices of SIYP, as reported in other studies ^27, 31^.

This study has some limitations. The main limitation is that our findings result from a qualitative approach; however, knowledge gaps in the use of contraceptives are best determined from a quantitative study. The study sought to explore SIYP perspectives of contraceptives and their use against the background of their sexual practices in South West; the findings may not represent other geo-political zones in Nigeria.

Despite the limitations, the findings of this study have highlighted that having access to accurate information on modern contraceptives and making available such modern contraceptives is of a priority for the SIYP. Most importantly, for the female SIYP, who are most vulnerable to high-risk pregnancies and unsafe abortion. Information about the benefits and risks of contraception is needful for informed decision-making which will help to prevent reproductive tract infections and sexually transmitted infections such as HIV/AIDS. The absence of these adverse health outcomes among SIYP will consequently improve their mental health and general well-being. Aside from contraceptive information that promotes safe sex among SIYP, the provision of other social safety net interventions, including cash transfer schemes and vocational training, would serve as alternatives to prostitution and transactional sex, thereby lessening the engagement in risky behaviour.

## Conclusion

This study concludes that SIYP are largely aware of contraception, especially the male condom. They are inconsistent with contraceptives and the low use of modern contraceptives is incongruent with their high-risk sexual practices. This may cause potential negative SRH outcomes for SIYP. The high-risk sexual practices SIYP engage in are mostly involuntary, due to their struggle for survival or coping with challenges on the street. Social safety net interventions for SIYP such as economic palliatives should be encouraged as alternatives to prostitution and transactional sex. Community sensitization and training of peer SIYP on contraceptive knowledge and use are recommended to dispel contraceptive myths, increase the uptake of contraceptives and promote safe sex practices.

## Data availability

### Underlying data

The qualitative data gathered and analysed in this study are not publicly available to protect the privacy of all participants. However, it can be made available from the corresponding author on reasonable request ( mobiyan@cartafrica.org) indicating research purpose and why raw data will be needed.

### Extended data

Figshare: EXTENDED DATA.docx. https://doi.org/10.6084/m9.figshare.14985177.v1 ^23^.

This project contains the following extended data:

- Focus group discussion guide
- In-depth interview guide
- Completed Consolidated criteria for reporting qualitative studies (COREQ) checklist

Data are available under the terms of the Creative Commons Attribution 4.0 International license (CC-BY 4.0).
