# Supplementary material for: The body cannot be cheated: sexual practices and modern contraceptive use among street-involved young people in two South West States in Nigeria
Source: AAS Open Res. 2021 Aug 4;4:41. [Version 1] doi: 10.12688/aasopenres.13241.1 (PMC11190648; doi:10.12688/aasopenres.13241.1)
Supplement: Peer review history

# Reviewer report: Reviewer response for version 1

**DOI:** 10.21956/aasopenres.14362.r31237

**Published:** 2024-06-20

**Authors:** Turnwait Otu Michael

**Affiliations:** 1 University of Johannesburg, Auckland Park, Gauteng, South Africa

Thank you for putting together this interesting research.

 The study explored the use of modern contraceptives and sexual practices among street-involved young people (SIYP) aged 10-24 years in Nigeria. The manuscript noted that despite high awareness of contraceptives, particularly condoms, usage remains low due to factors like reduced sexual pleasure, cost, and myths. The findings highlight the need for targeted interventions, including free contraceptive education and social safety nets, to mitigate high-risk sexual behaviors such as early sexual encounters, multiple sex partners, and transactional sex.

 However, below are some comments to improve the manuscript:

 Abstract:

 - Please provide more detail on the recruitment process for participants. You may include the sampling technique, depending on the word count allowed for the abstract.

 - Highlight key findings and their implications in a more structured way.

 - In the sentence, "Street-involved young people (SIYP) face a double burden of living condition instability and lack of adequate parental monitoring,” the phrase "living condition instability" would be clearer as "unstable living conditions."

 - In the sentence, "Both FGD discussions and IDI interviews were recoded; transcripts were transcribed and translated from local dialect into English language,” the word "recoded" should be "recorded," and "English language" should be "English."

 - The phrase "contraceptives pills" should be "contraceptive pills."

 - In the sentence, "The common reasons alluded for not using condoms were reduced sexual pleasure, cost and associated myths," "alluded for" should be "alluded to," and a comma should be added after "cost": "cost, and associated myths."

 - The phrase "transactional sex" is repeated and should only be mentioned once.

 Results Section:

 - Particularly in Theme 2: Perspectives and myths about modern contraceptives, the sentence "There were myths about contraceptives one of which is that condoms are only used by immoral people...” needs to be corrected for clarity. The current phrasing is unclear.

 Discussion Section:

 - The discussion lacks adequate references to past studies to show similarities and dissimilarities with previous research. This is important to highlight aspects of agreement and disagreement with previous studies.

 - The study should be guided by a theoretical framework.

 - Emphasize specific recommendations for policy and practice implications based on the study findings.

 - Address potential future research directions.

 - Improve the introduction/literature review with recent studies.

Is the work clearly and accurately presented and does it cite the current literature?

Partly

If applicable, is the statistical analysis and its interpretation appropriate?

Yes

Are all the source data underlying the results available to ensure full reproducibility?

Yes

Is the study design appropriate and is the work technically sound?

Yes

Are the conclusions drawn adequately supported by the results?

Yes

Are sufficient details of methods and analysis provided to allow replication by others?

Yes

Reviewer Expertise:

Demography, gender, sociology, sexual and reproductive health

I confirm that I have read this submission and believe that I have an appropriate level of expertise to confirm that it is of an acceptable scientific standard, however I have significant reservations, as outlined above.

# Reviewer report: Reviewer response for version 1

**DOI:** 10.21956/aasopenres.14362.r31229

**Published:** 2024-06-17

**Authors:** Onipede Wusu

**Affiliations:** 1 Lagos State University, Ojo, Lagos, Nigeria

Going through the paper, I observed it is a good report based on a qualitative investigation of sexual health behaviour among street-involved young people. The 17 IDIs and 11 FGDs generated rich data, and the analysis is fair. The findings indicated that risky sexual behaviours were common in the sample, but contraceptive use was poor.

 However, I have a few observations/suggestions that could improve the quality of the paper.

 Methods

 1. The study design should be restricted to design. Issues related to sampling, study guides and data collection should be moved to the study procedure section.

 2. How ethical was waving consent to participate in the study for the affected participants? The authors should provide more explanation on this question.

 3. The study population subsection is also overloaded with issues that should be in the study procedure subsection. The first two to three lines would suffice for the study population.

 Results

 4. I am uncomfortable with the use of percentages in the background characters of participants. It is better to use only numbers.

 5. I suggest the authors explore the analysis further to determine the frequencies of the themes in the data, show the numbers, and support the explanation with quotes. I think the NVIVo software makes this easy.

 I hope the authors find my suggestions useful.

Is the work clearly and accurately presented and does it cite the current literature?

Yes

If applicable, is the statistical analysis and its interpretation appropriate?

Not applicable

Are all the source data underlying the results available to ensure full reproducibility?

Yes

Is the study design appropriate and is the work technically sound?

Yes

Are the conclusions drawn adequately supported by the results?

Yes

Are sufficient details of methods and analysis provided to allow replication by others?

Partly

Reviewer Expertise:

Sexual and reproductive health

I confirm that I have read this submission and believe that I have an appropriate level of expertise to confirm that it is of an acceptable scientific standard, however I have significant reservations, as outlined above.

# Reviewer report: Reviewer response for version 1

**DOI:** 10.21956/aasopenres.14362.r28813

**Published:** 2021-08-24

**Authors:** Michelle Olivia Erasmus

**Affiliations:** 1 School of Public Health, University of Western Cape, Cape Town, South Africa

Thank you for this very relevant paper.

**Summary:**

 This qualitative study aimed to explore the perspectives of street-involved young people (SIYP) in Nigeria with regards to modern contraceptives. The authors used in-depth interviews as well as focus group discussions to gather data.

 The authors found that the sexual and reproductive health service needs of SIYP are largely unmet. There are also many myths and misconceptions regarding modern contraception, which leads to poor uptake of modern contraceptives amongst SIYP in Nigeria. Some of these myths are driven by societal norms and expectations regarding sexuality and same-sex relationships. Financial insecurity also has a major impact on access to modern contraception, e.g. condoms, as SIYP are not able to afford buying condoms. Some SIYP are financially dependent on others and therefore do not have decision-making power with regards to choice of contraception methods, and have to submit to the will of others in exchange for money, security, shelter or food.

 The authors recommend that social safety net interventions be put in place for SIYP, and that education programmes be tailored specifically to meet the needs of SIYP, such as peer training to dispel myths to about modern contraception, to improve uptake and promote safer sexual practices amongst SIYP.

**Feedback:**

- Abstract: The theme "transactional sex" appears twice under abstract.
- Results: Theme 3.2. Typo: does the author mean the age ranges, instead of raged?
- Table 1: total number of participants aged 20-24 is indicated as 1.3%, but should be 41.3% (possible typing error vs calculation error).
- Discussion: The authors mention that non-use of condoms are used as proof of infidelity. Isn't non-use of condoms rather used as proof of fidelity rather than infidelity? 

Thank you to the authors for this much-needed research.

Is the work clearly and accurately presented and does it cite the current literature?

Yes

If applicable, is the statistical analysis and its interpretation appropriate?

Not applicable

Are all the source data underlying the results available to ensure full reproducibility?

Yes

Is the study design appropriate and is the work technically sound?

Yes

Are the conclusions drawn adequately supported by the results?

Yes

Are sufficient details of methods and analysis provided to allow replication by others?

Yes

Reviewer Expertise:

Adolescent Sexual and reproductive health

I confirm that I have read this submission and believe that I have an appropriate level of expertise to confirm that it is of an acceptable scientific standard.
